# Effect of Melatonin on Rat Heart Mitochondria in Acute Heart Failure in Aged Rats

**DOI:** 10.3390/ijms19061555

**Published:** 2018-05-23

**Authors:** Irina Odinokova, Yulia Baburina, Alexey Kruglov, Irina Fadeeva, Alena Zvyagina, Linda Sotnikova, Vladimir Akatov, Olga Krestinina

**Affiliations:** 1 Institute of Theoretical and Experimental Biophysics, Russian Academy of Sciences, Pushchino, Moscow 142290, Russia; odinokova@rambler.ru (I.O.); byul@rambler.ru (Y.B.); krugalex@rambler.ru (A.K.); aurin.fad@gmail.com (I.F.); leavi@inbox.ru (A.Z.); linda_sotnikova@mail.ru (L.S.); akatov.vladimir@gmail.com (V.A.); 2 Pushchino State Natural Science Institute, Pushchino, Moscow 142290, Russia

**Keywords:** melatonin, permeability transition pore, rat heart mitochondria, acute heart failure, aging, ROS production, 2′,3′-cyclic nucleotide 3′-phosphodiesterase (CNPase), voltage-dependent anion channel (VDAC)

## Abstract

Excessive generation of reactive oxygen species (ROS) in mitochondria and the opening of the nonselective mitochondrial permeability transition pore are important factors that promote cardiac pathologies and dysfunction. The hormone melatonin (MEL) is known to improve the functional state of mitochondria via an antioxidant effect. Here, the effect of MEL administration on heart mitochondria from aged rats with acute cardiac failure caused by isoprenaline hydrochloride (ISO) was studied. A histological analysis revealed that chronic intake of MEL diminished the age-dependent changes in the structure of muscle fibers of the left ventricle, muscle fiber swelling, and injury zones characteristic of acute cardiac failure caused by ISO. In acute heart failure, the respiratory control index (RCI) and the Ca^2+^ retention capacity in isolated rat heart mitochondria (RHM) were reduced by 30% and 40%, respectively, and mitochondrial swelling increased by 34%. MEL administration abolished the effect of ISO. MEL partially prevented ISO-induced changes at the subunit level of respiratory complexes III and V and drastically decreased the expression of complex I subunit NDUFB8 both in control RHM and in RHM treated with ISO, which led to the inhibition of ROS production. MEL prevents the mitochondrial dysfunction associated with heart failure caused by ISO. It was shown that the level of 2′,3′-cyclicnucleotide-3′-phosphodiasterase (CNPase), which is capable of protecting cells in aging, increased in acute heart failure. MEL also retained the CNPase content in RHM both in control experiments and after ISO-induced heart damage. We concluded that an increase in the CNPase level promotes cardioprotection.

## 1. Introduction

Heart failure is a multifactorial clinical syndrome and a highly prevalent condition with an unfavorable prognosis [1]. Despite the fact that, over the past 20 years, effective methods of treatment have been developed, the mortality from heart failure remains extremely high, and the search for new approaches in the therapy of this disease is topical [2]. Mitochondria play a key role in the normal functioning of the heart, as well as in the pathogenesis and development of various heart diseases [3]. Physiologically, the stocks of mitochondrial ATP are consistent with changes in the ATP consumption by the heart, and mitochondrial Ca^2+^-transport routes that provide an increase in the mitochondrial concentration of Ca^2+^ mediates these changes [3]. In turn, this activates dehydrogenase enzymes to increase NADH and, consequently, the content of ATP. Pathologically, mitochondrial Ca^2+^ is important for generation of reactive oxygen species (ROS) and mitochondrial permeability transition pore (mPTP) opening, the factors involved in the onset of both ischemia–reperfusion and heart failure [3]. Because the mitochondrial Ca^2+^ content tends to decrease with aging, impaired mitochondrial Ca^2+^ handling and reduced threshold Ca^2+^ concentration may be critical in mediating the mPTP opening in aging. The regulators/modulators of mPTP, such as the voltage-dependent anion channel (VDAC) [4] and 2′,3′-cyclicnucleotide-3′-phosphodiasterase (CNPase) [5] are susceptible to oxidative damage in aging [6]. To reduce the age-dependent oxidative damage and mitochondrial dysfunction, much attention has been given to research aimed at increasing the protection against oxidative stress by antioxidants [7].

Melatonin (MEL) is a hormone synthesized by the pineal gland. It is a highly lipophilic molecule that penetrates through cell membranes, easily reaching subcellular structures [8]. In cells, MEL is found in nuclei and mitochondria [7,9]. In the course of life, the level of MEL changes. In young children, nocturnal MEL levels are the highest, approximately 325 pg·mL^−1^, and these decline gradually [10]. In older persons, MEL peak concentrations and total MEL production can reduce [11,12], although a great interindividual variability exists [13]. Rikie M. Scholtens et al. revealed that maximum concentrations of MEL varied greatly from 11.2 to 91.3 pg·mL^−1^. The maximum MEL level in studies with participants aged about 65–70 years was 49.3 pg·mL^−1^ and in studies with participants aged about ≥ 75 years was 27.8 pg·mL^−1^, *p*-value < 0.001 [14].

In the process of aging, the level of MEL in rat liver mitochondria decreases [15] and, consequently, its protective properties can be attenuated. It should be noted that chronic administration of MEL in experiments on animal models decreases blood pressure, reduces myocardial infarction and postinfarction remodeling and has antifibrotic effects on hearts of hypertensive rats [16,17,18,19]. Chronic treatment with MEL in a pharmacological dose affects the mitochondrial function and prevents mitochondrial dysfunction in experimental diabetes and after intoxication, demonstrating the mitochondrion-specific activity of MEL [20,21,22,23].

Recently, CNPase has been identified in rat brain and liver mitochondria, and it was shown that CNPase protects mitochondria from mPTP opening [5]. It is known that CNPase (CNP, EC 3.1.4.37) catalyzes the hydrolysis of 2′,3′-cyclic nucleotides to form the corresponding 2′-monophosphates [24]. To date, the functions of CNPase in mitochondria are poorly understood. The substrates of CNPase, 2′,3′-cAMP, and 2′,3′-cNADP were found to be able to enhance Ca^2+^-activated and CsA-sensitive pore opening in rat brain and liver mitochondria [5]. The changes in the CNPase level in mitochondria correlated with the activation of mPTP opening during aging [6]. Moreover, we demonstrated potential interaction of CNPase with regulators/modulators of mPTP, such as the VDAC, an adenine nucleotide translocator, and cyclophilin D in mitochondria [25]. Besides, we observed that CNPase and VDAC levels decreased with aging [6]. Later, we showed that MEL and CNPase were interrelated in mitochondria in such a way that MEL retained CNPase inside mitochondria to protect cells from damage [15]. We obtained CNPase in rat heart mitochondria (RHM) and showed that the CNPase level decreased in RHM in aging [26].

The aim of our research was to investigate the effect of MEL on the functional state of heart mitochondria of old rats with acute heart failure caused by isoprenaline hydrochloride (ISO) upon mPTP opening and to analyze changes in the levels of respiratory chain complexes, VDAC, and CNPase content in these conditions.

## 2. Results

### 2.1. Histological Analysis of Cryosections of the Left Ventricle of Rat Heart

**Figure 1:**
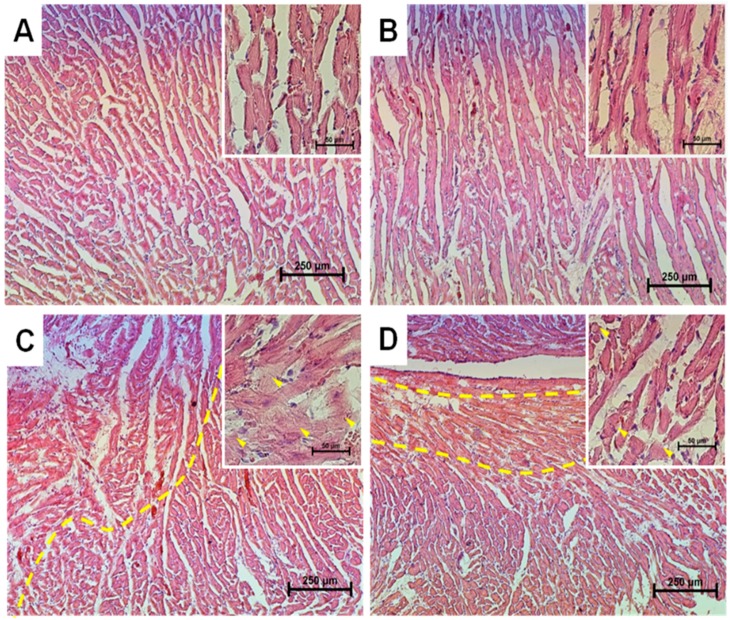
Histological preparations of the left ventricles from different groups of rats. (**A**) Control animals; (**B**) animals treated with melatonin (MEL) (~7 mg/kg of body weight per day); (**C**) animals treated with isoprenaline hydrochloride (ISO) (85 mg/kg of body weight twice); (**D**) animals treated with MEL + ISO. The yellow stroke restricts the area of degenerative changes. The foci of granular dystrophy are indicated by yellow arrows.

A histological analysis of cryosections of the left ventricle of rat heart showed that the samples of the control (Group 1) corresponded to the standard parameters of the structure and architectonics of the myocardium of a particular age group of Wistar rats (Figure 1A). Tissue samples from rats of Group 2, which received MEL for two months, markedly differed from the control; namely, the structure of muscle fibers was better preserved, and age-related changes were less pronounced (Figure 1B). In tissue samples from rats of Group 3 (two successive injections of ISO), a significant degeneration of the myocardium was observed (46.2 ± 7.37%), which made itself evident in the swelling of muscle fibers, complete blurring of myofibrillar contours, the vacuolization of the sarcoplasm, grain dystrophy, the appearance of loci of myolysis with lumpy decomposition of fibers, as well as the development of small focal necrosis, with micromalacia (up to the disappearance) of cell nuclei.

In samples from rats of Group 4 (MEL + ISO), partial degenerative changes in the myocardium were observed (23.76 ± 6.28%), which manifested themselves in small focal homogenization of myofibrils with the smoothing (up to the complete disappearance) of cross-striation and the loss of intramyofibrillar contours, as well as the appearance of local vacuolization of the sarcoplasm without marked swelling of muscle fibers (Figure 1D). The absence of marked swelling of muscle fibers and demarcation localization of the zone of injury induced by the injection of ISO indicated the protective action of MEL.

### 2.2. Respiratory Control Index (RCI) of RHM

**Figure 2:**
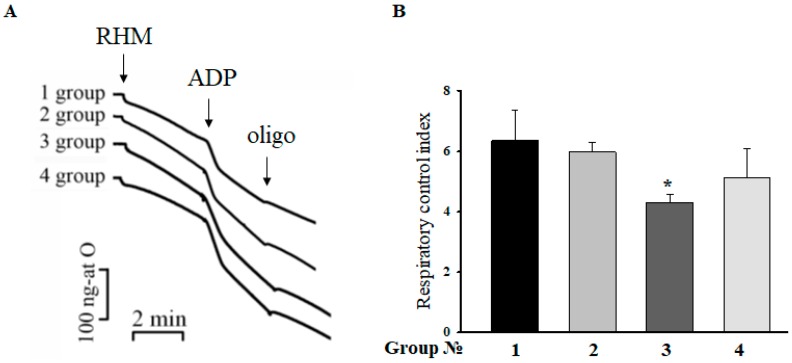
Respiratory control index of mitochondria isolated from different group of rats. Panel (**A**) The curves of respiratory activity of rat heart mitochondria (RHM). The arrows show the times of addition of 1 mg/mL RHM, 200 µM ADP (adenosine diphosphate), and 1.5 µM oligomycin, respectively. Panel (**B**) A diagram reflecting the quantitative analysis of the respiratory control index (RCI). The values shown are the means ± SD from three independent experiments. * *p* < 0.05 significant difference in the protein level in comparison with the corresponding control.

The RCI indicates the effectiveness of mitochondria in promoting oxidative phosphorylation. Therefore, at the next step, we measured the RCI of mitochondria isolated from each group of rats. Figure 2A demonstrates the curves of mitochondrial respiration under the conditions used. The values of RCI in different states are given in Figure 2B. According to the recent data, no substantial changes in RCI were observed as a function of aging, indicating that the mitochondrial membrane remains essentially unaffected by age [15]. On the contrary, the RCI in rats injected with ISO decreased by 30%, whereas after the administration of MEL in combination with ISO, the RCI in RHM remained unchanged compared with the control.

### 2.3. Effect of MEL and ISO on the Level of Enzymes in the Electron Transport Chain in RHM

**Figure 3:**
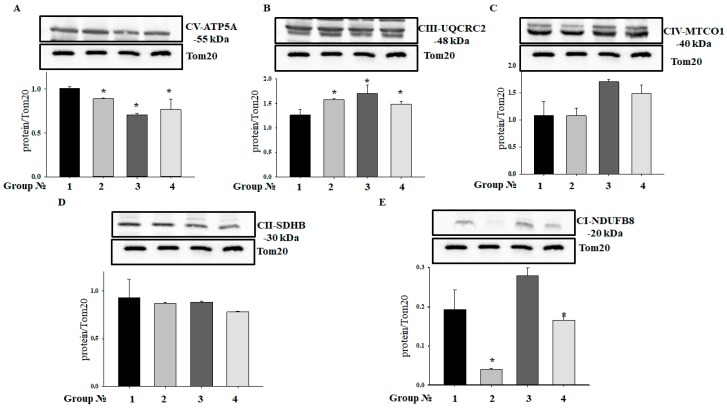
Alterations in mitochondrial respiratory chain complexes in RHM. Protein samples were extracted and subjected to Western blotting. Changes in mitochondrial complexes were detected using the Total OXPHOS Rodent WB Antibody Cocktail. (**A**): changes in complex V alpha subunit (CV-ATP5A-55 kDa), (**B**): complex III core protein 2 (CIII-UQCRC2-48 kDa), (**C**): complex IV subunit I (CIV-MTCO1-40 kDa), (**D**): complex II subunit 30 (CII-SDHB-30 kDa), (**E**): complex I subunit NDUF88-20 kDa (CI-NDUFB8). Immunodetection of Tom20 was used as a loading control. The upper parts of panels represent immunostaining with an OXPHOS antibody cocktail and Tom20. The lower parts show the quantification of immunostaining using computer-assisted densitometry. Bar graphs represent the levels of appropriate complexes with respect to the Tom20 level in absolute units. The data are presented as the means ± SD. * *p* < 0.05 significant difference in the protein level in comparison with the corresponding control.

Acuna-Castroviejo and coworkers showed that MEL improved the activity of the electronic transport chain (ETC), reducing the ROS formation in I and IV complexes [27]. We determined changes in the levels of enzymes in the ETC, which may reflect changes in their activity in RHM isolated from each group of rats (Figure 3). Figure 3A shows changes in the level of the complex V (CV) alpha subunit. We observed that treatment with MEL decreased the CV alpha subunit level by 11.6% (RHM from Group 2 vs. RHM from Group 1), while ISO diminished the CV alpha subunit level by 30% (RHM from Group 3 vs. RHM from Group 1). The combined action of MEL and ISO led to a decline in the CV alpha subunit level by 23% (RHM from Group 4 vs. RHM from Group 1). The alterations in the CIII core protein 2 level in RHM in each experimental group are shown in Figure 3B. MEL increased the CIII core protein 2 level in RHM by 25%, while ISO and ISO in combination with MEL upregulated the CIII core protein 2 level in RHM by 35% and 18%, respectively. We did not notice any changes in the levels of CIV and CII subunits in RHM isolated from Groups 2, 3, and 4 in comparison with RHM from Group 1. On the contrary, MEL treatment diminished the CI subunit level by 80%, and MEL in combination with ISO decreased it by 14%.

### 2.4. Effect of the MEL, ISO, and the Combined Effect of MEL and ISO on Changes in the Calcium Retention Capacity

**Figure 4:**
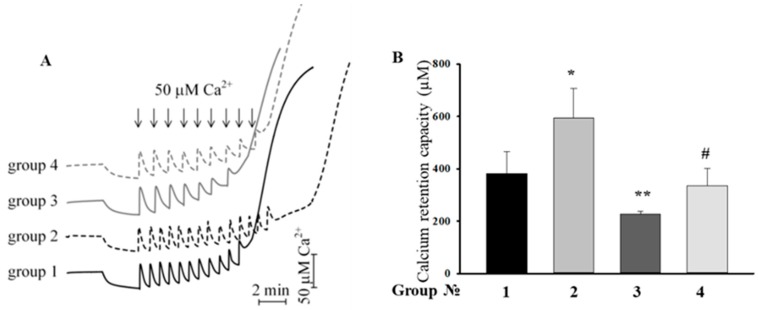
Comparison of the calcium retention capacity of mitochondria isolated from different groups of rats. Group 1: Control animals, Group 2: animals treated with MEL (~7 mg/kg of body weight per day), Group 3: animals treated with ISO (85 mg/kg of body weight twice), Group 4: animals treated with MEL + ISO. (**A**) Records of Ca^2+^ fluxes in RHM. Arrows show the times at which CaCl_2_ (50 nmol of Ca^2+^ per mg of protein) was applied; (**B**) Quantitative analysis of Ca^2+^ retention capacity. The values shown are the means ± SD from three independent experiments. * *p* ≤ 0.05, ** *p* ≤ 0.01 vs. the Ca^2+^ retention capacity of mitochondria from control rats (Group 1), # *p* ≤ 0.05 compared with the Ca^2+^ retention capacity of mitochondria from animals treated with ISO (Group 3).

Then, we examined the influence of MEL and ISO on the mPTP functioning of RHM. Calcium pulses (50 µM each) were added to mitochondria to reach a threshold calcium concentration for mPTP opening. In RHM from each experimental group, the first addition of Ca^2+^ led to an active accumulation of Ca^2+^ in mitochondria with subsequent restoration (Figure 4A). The release of accumulated Ca^2+^ (mPTP opening) occurred after the 11th, 14th, 7th, and 9th Ca^2+^ addition in RHM from control animals and from rats treated with MEL, ISO, and MEL+ISO, respectively. Figure 4B demonstrates quantitative changes in the Ca^2+^ retention capacity of Ca^2+^-loaded mitochondria in RHM isolated from each experimental group of rats. We observed that MEL increased the Ca^2+^ retention capacity by 55%, while ISO decreased it by 40%. After the combined action of MEL and ISO, the Ca^2+^ retention capacity did not change compared with the control (RHM from Group 1), whereas compared with RHM from Group 3, it increased by 47%. MEL abolished the inductive effect of ISO in RHM.

### 2.5. Effect of MEL and ISO on the Swelling of RHM

**Figure 5:**
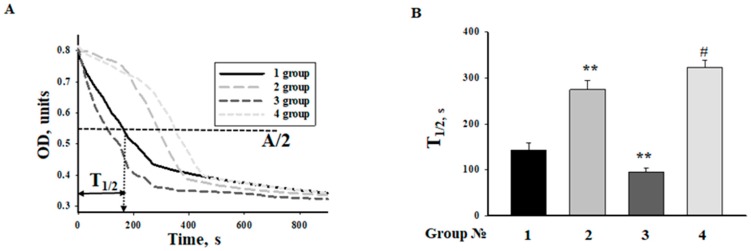
Ca^2+^-dependent swelling of RHM from rats of each group upon mPTP opening. RHM were incubated in standard medium as described in Methods. Group 1: Control animals, Group 2: animals treated with MEL (~7 mg/kg of body weight per day), Group 3: animals treated with ISO (85 mg/kg of body weight twice), Group 4: animals treated with MEL + ISO. (**A**) Curves of RHM swelling. (**B**) Average half-times (T_1/2_) of swelling. T_1/2_ was calculated as shown on Panel A. ** *p* ≤ 0.01 vs. the T_1/2_ of mitochondria from control rats (group 1), # *p* ≤ 0.05 compared with the T_1/2_ of mitochondria from animals treated with ISO (group 3).

Calcium-induced mitochondrial swelling is one of the characteristics of the mPTP opening. The addition of Ca^2+^ at the threshold concentration to a mitochondrial suspension incubated in the standard medium caused a decrease in light scattering, which is indicative of swelling. We compared the swelling of RHM isolated from each group of rats. Figure 5A shows the representative curves of Ca^2+^-activated swelling of RHM isolated from rats of four groups. Figure 5B demonstrates the average half-time (T_1/2_) of mitochondrial Ca^2+^-activated swelling. The half-time of swelling of mitochondria in rats treated with MEL increased by 90%. Thus, the rate of swelling of RHM became slower as compared to the mitochondrial swelling in control RHM. The half-time of mitochondrial swelling in rats treated with ISO decreased by 34%, while the time needed for the swelling of RHM by the combined action of ISO and MEL increased 2-fold. ISO accelerated mitochondrial swelling, whereas MEL abolished the effect of ISO.

### 2.6. Effect of MEL and ISO on ROS Production upon mPTP Opening in RHM

**Figure 6:**
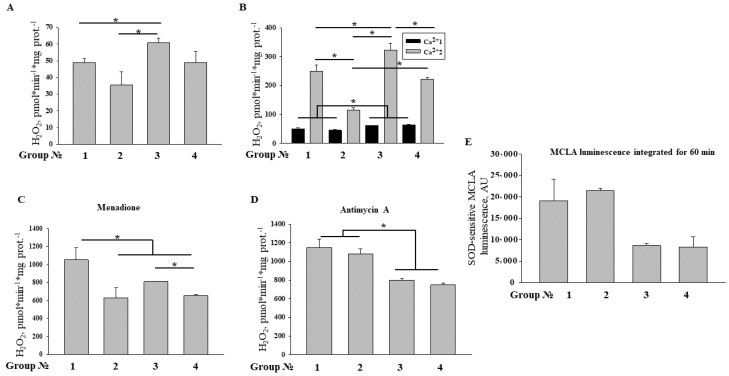
Reactive oxygen species (ROS) production in RHM from control animals and animals treated with MEL/ISO. RHM (0.33 mg/mL) were placed in the incubation medium supplemented with 5 mM glutamate, 5 mM malate, 20 µM Amplex Red, horseradish peroxidase (HRP) (3 U/mL), and 10 µM EGTA (Panels **A**–**D**), and transferred to wells, which contained, where indicated, 100 µM Ca^2+^ (**B**), 5 µM menadione (**C**), and 2 µM antimycin A (**D**). (**E**) Production of superoxide anion (SA) in RHM from control animals and animals treated with MEL/ISO. RHM (0.33 mg/mL) were placed in the incubation medium supplemented with 5 mM glutamate, 5 mM malate, 20 µM MCLA, and 10 µM EGTA. Some samples contained superoxide dismutase (SOD, 100 U/mL) for the calibration of luminescent response. The panel shows a SOD-sensitive part of the chemiluminescent signal integrated for 60 min of incubation. Values in columns are the means ± SD for three independent experiments for each group (*n* = 9). * *p* < 0.05, significant difference in comparison with the corresponding control.

Then, we assessed the ROS production by RHM isolated from control animals and animals treated with MEL/ISO (Figure 6). In the presence of glutamate and malate, RHM from control, MEL, and ISO-treated rats produced H_2_O_2_ with rates of ~50, 35, and 62 pmol·min^−1^·mg protein^−1^, respectively (Figure 6A). The production of H_2_O_2_ by RHM from MEL plus ISO-treated animals was at the control level. It is well known that Ca^2+^ accelerates ROS production in mitochondria via the activation of matrix NAD(P)-dependent dehydrogenases and mPTP opening. We measured the rates of H_2_O_2_ production in RHM immediately (Ca^2+^1) and 45 min after (Ca^2+^2) the addition of 100 µM Ca^2+^ (Figure 6B). As it follows from the figure, in RHM from ISO-treated animals, H_2_O_2_ generation immediately after the addition of Ca^2+^ was ~15% greater than in the control, and treatment with MEL did not reduce it. After 45 min of incubation, the production of H_2_O_2_ in RHM from ISO- and MEL-treated rats was ~30% higher and 2.5 times lower than in the control, respectively, which, presumably, is related to the acceleration and inhibition of mPTP opening, respectively (Figure 4 and Figure 5). MEL reduced the H_2_O_2_ generation in RHM damaged by ISO to the control level. Redox-cycling menadione is known to accept electrons from complex I and diaphorase and to transfer them to oxygen, generating ROS. Figure 6C shows that menadione-induced H_2_O_2_ generation in RHM (~1000 pmol·min^−1^·mg protein^−1^) was lowered by treatment with MEL and ISO by 40% and 20%, respectively, and that the effects of MEL and ISO were not additive. By contrast, antimycin A-dependent H_2_O_2_ production in complex III was not affected by MEL but was suppressed by ISO by 20–30% (Figure 6D). The release of superoxide anion (SA) from intact RHM (Figure 6E) was also suppressed in ISO-treated rats and treatment with MEL did not recover it. These data suggest that both MEL and ISO are capable of affecting the respiratory complex I, while complex III can be affected only by ISO. Moreover, treatment with MEL and ISO causes opposite effects on the mPTP opening and hence ROS production.

### 2.7. The Effect of MEL and ISO on the Levels of CNPase and VDAC in RHM

**Figure 7:**
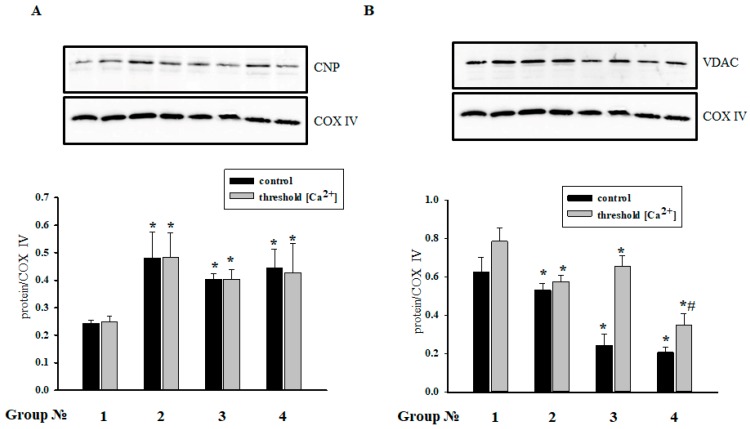
Alterations in the levels of 2′,3′-cyclic nucleotide 3′-phosphodiesterase (CNPase (**A**)) and voltage-dependent anion channel (VDAC (**B**)) in RHM from different groups of rats. Group 1: control animals, Group 2: animals treated with MEL (~7 mg/kg of body weight per day), Group 3: animals treated with ISO (85 mg/kg of body weight twice), Group 4: animals treated with MEL + ISO. Protein samples were extracted and subjected to Western blotting. Immunodetection of COX IV was used as a loading control. The **upper** parts of panels—the immunostaining of CNPase, VDAC and COX IV. The **lower** parts—quantification of immunostaining using computer-assisted densitometry. Bar graphs represent the levels of appropriate proteins in absolute units. The data are presented as the means ± SD. * *p* < 0.05 significant difference in protein level in RHM from Groups 2–4 vs. the corresponding control of Group 1 (black bars for samples without Ca^2+^, gray bars for samples with Ca^2+^), # *p* ≤ 0.05 compared with the protein level of mitochondria from animals treated with ISO (Group 3).

We assumed that CNPase is capable of protecting cells in aging [6]. It was found that CNPase coprecipitates with VDAC [25], and MEL is involved in the preservation of CNPase within mitochondria [15]. In the present study, we determined the contents of CNPase and VDAC in RHM in the experimental conditions used (Figure 7). Figure 7A,B (upper parts) show Western blots of the CNPase and VDAC levels in RHM isolated from each group of rats. A quantitative analysis of CNPase and VDAC levels is shown in Figure 7A,B (lower parts). Protein bands were quantified after normalization with respect to COX IV. After treatment with MEL, the CNPase level increased 2 times in RHM when mPTP was closed/opened (Group 2), and after injection of ISO, the CNPase content increased 1.7 times when mPTP was closed/opened (Group 3) in comparison with control RHM (Group 1). ISO did not change the CNPase level in MEL-treated rats (Group 4) in comparison with RHM from the Group 3. 

On the contrary, after MEL treatment, the VDAC content in RHM decreased when mPTP was opened, but it did not change compared with the control when mPTP was closed (Group 1). The VDAC level dramatically diminished in RHM of rats injected with ISO (Group 3) 2.6 times when mPTP was closed, but it did not change compared with the control when mPTP was opened. Added ISO did not affect the VDAC level in RHM of MEL-treated rats (Group 4) in comparison with RHM from Group 3 of rats when mPTP was closed, and the VDAC level decreased 3 times compared with the control (Group 1); the VDAC level decreased 2.2 times as compared with the control and 1.85 times compared with the VDAC content in RHM from Group 3 of rats when mPTP was opened.

## 3. Discussion

Heart failure is one of the main causes of morbidity and mortality worldwide and a timely problem in elderly people [28]. Hypertension, coronary artery disease, uncompensated hypertrophy, inherited or acquired valvular disease, cardiomyopathy, and other conditions lead to cardiac insufficiency [29]. It has been long known that mitochondrial dysfunction (i.e., the inability to produce ATP in amounts sufficient for cellular demands) is critical for the development of the disease [29]. Mitochondrial dysfunction develops with aging. Its development is associated with oxidative stress, a decreased activity of the calcium-transporting mitochondrial system [30,31], and an increase in the nonselective permeability of the inner membrane (mPTP opening due to an elevated concentration of calcium ions in the matrix) [32,33]. Recently, we showed that mitochondria from aged animals display an enhanced susceptibility to the mPTP opening, which makes itself evident as by decrease in the threshold calcium concentration and acceleration of mPTP opening [6,34]. Moreover, in various models of heart failure, except for compensated hypertrophy, numerous defects in the electron transport chain complexes have been described [35]. It should be noted that defects in respiratory complexes and FoF_1_-ATPase have a dual effect on the mitochondrial dysfunction. On the one hand, they diminish the ATP production, which is critical for the maintenance of cytosolic Ca^2+^ at a low level. On the other hand, the inhibition of respiratory complexes causes the accumulation of reducing equivalents (NAD(P)H), which suppress both substrate oxidation and mPTP opening [36].

In the present study, we used a model of the acute heart failure caused by ISO (Figure 1) with pronounced degeneration of the striated muscle of the left ventricle [37]. The study of morphological parameters did not reveal any significant changes in the weight of the left and right ventricles of rats; however, in the experimental group (Group 3), diffuse necrotic zones were observed (data not shown). The experiments on this model revealed a partial mitochondrial respiration uncoupling (Figure 2) and enhanced mitochondrial susceptibility to mPTP opening (Figure 4 and Figure 5), as well as a decrease in the CV-ATP5A level by 30% (Figure 3A) and, probably, in the activity of complex V in isolated RHM. At the same time, the level of CIII-UQCRC2 increased by 35%, which was accompanied by a 30% decrease in the antimycin A-dependent ROS production and a considerable decline in the release of superoxide from intact RHM (Figure 6D). Antimycin A-dependent ROS production shows the maximum possible ROS generation in the outer quinone-binding center of complex III [38]. Thus, the complex III subunit may be upregulated in order to reimburse the complex III deficiency in rats treated with ISO. Therefore, the acceleration of ROS production caused by ISO treatment in the absence of antimycin A (Figure 6A,B) was, presumably, due to the facilitation of mPTP opening (see Figure 4 and Figure 5) but not due to the damage to mitochondrial complexes I and III. 

The protective effect of MEL against the development of the mitochondrial dysfunction can be related to the inhibition of mPTP opening and release of cytochrome *c* in RHM [9,39]. Earlier, we showed that long-term treatment with MEL suppresses the opening of the mPTP (increases the threshold Ca^2+^ concentration and decelerates the pore opening) in brain mitochondria isolated from aged animals [40] and prevents the cumene hydroperoxide-induced swelling of brain mitochondria isolated from young and aged rats. We concluded that MEL abolishes the pro-oxidant effect of cumene hydroperoxide [41]. 

It is known that chronic treatment with MEL at a pharmacological dose affects mitochondrial function [42,43]. Here, we showed that MEL drastically decreased the expression of complex I subunit NDUFB8 (Figure 3), which was accompanied by the suppression of ROS production in the absence and in the presence of the redox cycler menadione (Figure 6A–C) and the inhibition of mPTP opening (Figure 4 and Figure 5). The menadione-dependent ROS production reflects the NAD(P)H–quinone oxidoreductase activity in the initial segment of the mitochondrial respiratory chain [44]. Therefore, the suppression of ROS production by MEL was, most likely, due to both the downregulation of complex I and inhibition of mPTP opening. It is noteworthy that the preservation of matrix NAD(P)H in a reduced state (via complex I inhibition) precludes the mPTP opening by Ca^2+^ [36]. 

According to our data, MEL antagonizes the mitochondrial dysfunction associated with heart failure caused by ISO due to mPTP inhibition. The role of oxidation of substrates donating electrons to the respiratory chain regions that follow complex I deserves further elucidation. 

The results of this study are consistent with the previously obtained data indicating that long-term treatment with MEL detained mPTP opening in RHM. In our experiments, ISO induced mPTP opening in RHM and hence decreased the Ca^2+^ retention capacity by 40%, whereas MEL prevented the effect of ISO and increased the Ca^2+^ retention capacity. MEL displayed a protective effect in RHM isolated from ISO-injected rats.

Recently, we have found CNPase in the outer and inner membranes of rat brain and liver mitochondria [5,45]. We noticed that CNPase participated in the regulation of mPTP [5] and coprecipitated with mPTP regulators/modulators, such as VDAC, ANT, and cyclophilin D [25]. In aging, VDAC is susceptible to oxidative damage [46]. We showed that the VDAC level in rat brain mitochondria decreased with aging [6]. Moreover, we revealed that CNPase is associated with respiratory chain complexes in the inner membrane of rat brain, liver, and heart mitochondria, and the level of CNPase diminishes in V, III, and I complexes in rat brain mitochondria upon mPTP opening [25]. Whether the protein is involved in cardioprotection is still unknown. Our results showed that the level of CNPase increased in acute heart failure, probably to protect mitochondria from damage, and MEL strengthened this effect. The content of VDAC decreased when mPTP was closed in acute heart failure (Group 3) and MEL did not abolish the effect (Group 4). Because VDAC and CNPase coprecipitated, we propose that CNPase might compensate for VDAC modulation in mitochondria. The mechanisms of mitochondrial protection by MEL upon the ISO-induced heart failure will be elucidated in our further research.

## 4. Materials and Methods

### 4.1. Animal and Treatment

Twelve aged male Wistar rats (12-months old) used in the experiment were divided into four groups. For each separate experiment, one rat was used; thus, three independent replicates were done for each experimental group. Animals were individually housed in a temperature-controlled room (22 °C) and kept on a standard diet with full access to water and food. The rats of Group 1 and Group 3 received normal drinking water. The rats of Group 2 and Group 4, starting from an age of 10 months, received MEL (Sigma, St. Louis, MO, USA) (100 μg/mL) dissolved in drinking water for 2 months. The volume of daily water intake was 33 ± 3 mL per rat, which made up approximately 7 mg of MEL per kg of body weight per day [47]. After 2 months, the rats of Groups 3 and 4 were injected with ISO dissolved in physiological saline (85 mg/kg of body weight,) twice at an interval of 24 h to induce acute heart failure [37]. Animals of Group 1 (control) and Group 2 (treatment with MEL) were injected subcutaneously with saline (as a vehicle). Rats were sacrificed by cervical dislocation 24 h after the second ISO or saline injection at the same time, at 10 a.m. Hearts were removed, cleared of blood, and immediately transferred to ice-cold containers with 0.9% sodium chloride. All experiments were performed in accordance with the “Regulations for Studies with Experimental Animals” (Decree of the Russian Ministry of Health of 12 August 1997, No. 755). The protocol was approved by the Commission on Biological Safety and Ethics of the Institute of Theoretical and Experimental Biophysics, Russian Academy of Science (November 2014, protocol N45). 

### 4.2. Histological Analysis

To prevent autolysis, immediately after the withdrawal of the heart (within 10 min after decapitation), fragments of the left ventricle were washed for 30 s with cold (+14 °C) physiological saline and fixed with neutral buffered formalin (NBF) for 24 h at the tissue volume to fixative volume ratio of 1:30. After the termination of fixation, ventricle fragments were washed with distilled water (for 15 s each time) to remove phosphates and placed for at least 1 h in embedding medium O.С.T. (Optimum Cutting Temperature) Compound Tissue Tek (Sakura, Tokyo, Japan). Then, 9 μm wide sections of rat heart ventricles were prepared by a Shandon Cryotome 620E (Thermo Fisher Sci., Waltham, MA, USA). H&E (Mayer’s Hematoxylin and Eosin Y) staining of samples was carried out as described in [48]. Microphotographs of stained histological samples were obtained and processed using a Nikon Eclipse Ti-E microscope (Nikon, Tokyo, Japan) and the NIS-Elements software (Nikon, Tokyo, Japan).

### 4.3. Isolation of Rat Heart Mitochondria

Mitochondria were isolated from the heart of old (12-months old) male Wistar rats by the standard technique [49]. The isolated heart was chopped, cleared from blood vessels, and destroyed with a glass homogenizer in a 10-fold volume of the medium containing 75 mM sucrose, 10 mM Tris-HCl (pH 7.4), 225 mM mannitol, 1 mM EDTA, and 0.1% BSA at 4 °C. The homogenate was centrifuged at 1000× *g* for 10 min, and the pellet was removed. The mitochondria contained in the supernatant were sedimented at 6000× *g* for 10 min at 4 °C. Then, the mitochondrial pellet was washed with the isolation medium without EDTA and BSA (6000× *g*, 10 min) and suspended in the same medium. The protein content in mitochondria was determined using the Bradford assay. Protein concentration in a rat heart mitochondrial suspension was 30–35 mg/mL. Isolated mitochondria were kept for 2 h at 4 °C.

### 4.4. Evaluation of Mitochondrial Functions

The Ca^2+^ retention capacity of RHM was determined with a Ca^2+^-sensitive electrode (Nico, Moscow, Russia), and the oxygen consumption rate was measured with a Clark-type O_2_ electrode in a 1 mL measuring chamber [50]. Mitochondria (1 mg protein/mL) were incubated in a medium containing 125 mM KCl, 10 mM Tris (pH 7.4), 2 mM K_2_HPO_4_ at 25 °C. Glutamate (5 mM) and malate (5 mM) were used as respiratory substrates. The respiratory control index (RCI) was measured in a closed chamber after the addition of 200 μM ADP. The RCI of mitochondria from rats of Group 1 was used as a control and was taken to be unity. The mPTP opening in RHM was induced by a threshold Ca^2+^ concentration (each addition of Ca^2+^ contained 50 μM). A threshold Са^2+^ concentration was defined as that concentration of Ca^2+^ added to a mitochondrial suspension at which Ca^2+^ ions (when accumulating in mitochondria) induce the mPTP opening.

The swelling of RHM was estimated by measuring a change in light scattering in a mitochondrial suspension at 540 nm (A540) using a Tecan Infinite 200 spectrophotometer at 25 °C. The standard incubation medium for the swelling assay contained 125 mM KCl, 10 mM Tris (pH 7.4), 2 mM KH_2_PO_4_, 5 mM glutamate, and 5 mM malate. The concentration of the mitochondrial protein in a well was 0.5 mg protein/mL. Swelling was initiated by the addition of 300 nmol of Ca^2+^ per mg of protein. The swelling process was characterized by the time needed to reach the half-maximal light-scattering signal (T_1/2_).

### 4.5. ROS Measurement in Heart Mitochondria

The level of hydrogen peroxide in a mitochondrial suspension was measured as described previously [51] in standard medium supplemented with 5 mM glutamate, 5 mM malate, 20 µM Amplex Red, horseradish peroxidase (HRP) (3 U/mL), 10 µM EGTA, and, where indicated, 100 µM Ca^2+^, 5 µM menadione, and 2 µM antimycin A. Resorufin accumulation was traced using a plate fluorimeter (Tecan Infinite 200) in 96-well plates at excitation and emission wavelengths of 530 and 595 nm. For the quantitative assessment of hydrogen peroxide, fluorescence was calibrated by an excess of hydrogen peroxide at the end of measurements. In order to avoid light-induced resorufin formation, the fluorescence was measured one or two times a minute.

The rate of SA production was assessed using the highly sensitive chemiluminescent probe 3.7-dihydro-2-methyl-6-(4-methoxyphenyl)imidazo[1,2-a]pyrazine-3-one (MCLA) [52]. The kinetics of MCLA-derived chemiluminescence (MDCL) was recorded using a plate reader (Tecan Infinite 200) in accordance with a specified protocol [53]. Some samples contained superoxide dismutase (100 U/mL) for the calibration of luminescent response. Other experimental details are given in figure legend.

### 4.6. Sample Preparation, Electrophoresis, and Immunoblotting of Mitochondrial Proteins

To prepare samples for the determination of the level of OxPhos Complexes, the aliquots (2 mg/mL) of native RHM were placed in an Eppendorf tube and solubilized in Laemmli buffer. The samples were heated to 37 °C for 3 min. 

To determine the level of mitochondrial proteins, 50 μL aliquots were taken from the chamber, placed in an Eppendorf tube, and Laemmli buffer was added to solubilize mitochondrial proteins. The samples were heated to 95 °С for 5 min and applied to the gel. 

Samples containing 30 μg of mitochondrial protein were applied to each lane and subjected to electrophoresis followed by Western blot analysis. Mitochondrial samples were separated by 12.5% SDS-PAGE and transferred to a nitrocellulose membrane at 300 mA for 1 h. Precision Plus Pre-stained Standards from Bio-Rad Laboratories (Hercules, CA, USA) were used as markers. 

Alterations in the levels of ETC enzymes were detected with Total Oxphos Rodent WB Antibody Cocktail (ab 110413, monoclonal antibodies). The Oxphos Antibody Cocktail consists of complex V alpha subunit (CV-ATP5A-55 kDa), complex III core protein 2 (CIII-UQCRC2-48 kDa), complex IV subunit I (CIV-MTCO1-40 kDa), complex II subunit 30 (CII-SDHB-30 kDa), complex I subunit NDUF88-20 kDa (CI-NDUFB8). The Tom20 antibody (1:1000 dilution; Cell Signaling, Danvers, MA, USA) was used as a loading control.

The monoclonal anti-CNP antibody (anti-CNP Ab) was obtained as described [54] and used at the 1:10,000 dilution, the VDAC antibody (1:1000) was purchased from Calbiochem, and the COX IV antibody (1:1000 dilution, Abcam, Cambridge, UK) was used as a loading control.

The immunoreactivity was detected using the appropriate secondary antibody conjugated to horseradish peroxidase (Jackson Immuno Research, West Grove, PA, USA). Peroxidase activity was detected with ECL (Bio-Rad, Hercules, CA, USA) using the ChemiDoc Touch Imaging System (Bio-Rad). Protein bands were quantified by densitometry (Image Lab program, Bio-Rad, Hercules, CA, USA).

### 4.7. Statistical Analysis

For statistical analysis, the relative levels of protein density were expressed as mean ± SD from at least three to five independent experiments. The statistical significance of the difference between the mean values was evaluated using the Student’s *t*-test. The difference was considered significant at *p* < 0.05.

## 5. Conclusions

Histological analysis showed the presence of acute heart failure induced by ISO, which manifested itself in the pronounced swelling of muscle fibers, complete blurring of myofibrillar contours, vacuolization of sarcoplasm, granular dystrophy, and the appearance of myolysis foci with a lumpy decomposition of fibers. MEL administration abolished the effect ISO, which indicates its protective action. In acute heart failure, the RCI and the Ca^2+^ retention capacity in isolated RHM decreased. MEL increased the RCI and Ca^2+^ retention capacity in RHM. MEL and ISO were capable of affecting the respiratory complex I, while complex III was affected only by ISO. Treatment with MEL and ISO caused opposite effects on the mPTP opening and ROS production. CNPase was able to protect cells in aging [6], and MEL was found to be involved in the preservation of CNPase within mitochondria [15]. In acute heart failure, the level of CNPase in RHM increased, while the VDAC level decreased. MEL preserved the CNPase content in RHM, probably for their protection from damage. We concluded that CNPase takes part in the development of cardioprotection. The mechanisms by which this occurs are to be established in our further studies and additional experiments are needed for understanding the multifunctional role of mitochondrial CNPase in heart diseases.

## Abbreviations

RHM	rat heart mitochondria
VDAC	voltage dependent anion cannel
CNPase	2′,3′-cyclic nucleotide-3′-phosphodiesterase
ROS	reactive oxygen species
mPTP	mitochondrial permeability transition pore
ISO	isoprenaline hydrochloride
MEL	melatonin
RCI	respiratory control index
ETC	electronic transport chain
